# Cell-based RNAi screening and high-content analysis in primary calvarian osteoblasts applied to identification of osteoblast differentiation regulators

**DOI:** 10.1038/s41598-018-32364-8

**Published:** 2018-09-19

**Authors:** Mubashir Ahmad, Torsten Kroll, Jeanette Jakob, Alexander Rauch, Aspasia Ploubidou, Jan Tuckermann

**Affiliations:** 1 Institute of Comparative Molecular Endocrinology (CME), Ulm University, Helmholtzstrasse 8/1, 89081 Ulm, Germany; 2 Leibniz Institute on Aging – Fritz Lipmann Institute (FLI), Beutenbergstrasse 11, D-07745 Jena, Germany

## Abstract

Osteoblasts are responsible for the maintenance of bone homeostasis. Deregulation of their differentiation is etiologically linked to several bone disorders, making this process an important target for therapeutic intervention. Systemic identification of osteoblast regulators has been hampered by the unavailability of physiologically relevant *in vitro* systems suitable for efficient RNAi and for differentiation read-outs compatible with fluorescent microscopy-based high-content analysis (HCA). Here, we report a new method for identification of osteoblast differentiation regulators by combining siRNA transfection in physiologically relevant cells with high-throughput screening (HTS). Primary mouse calvarial osteoblasts were seeded in 384-well format and reverse transfected with siRNAs and their cell number and differentiation was assayed by HCA. Automated image acquisition allowed high-throughput analyses and classification of single cell features. The physiological relevance, reproducibility, and sensitivity of the method were validated using known regulators of osteoblast differentiation. The application of HCA to siRNAs against expression of 320 genes led to the identification of five potential suppressors and 60 activators of early osteoblast differentiation. The described method and the associated analysis pipeline are not restricted to RNAi-based screening, but can be adapted to large-scale drug HTS or to small-scale targeted experiments, to identify new critical factors important for early osteoblastogenesis.

## Introduction

Understanding the molecular mechanism of osteoblast differentiation is essential for improvement of therapeutic approaches for bone-related pathological conditions including osteoporosis^1–5^. Most of the pharmacological agents used in osteoporosis treatment are antiresorptive drugs that stabilize bone mass, to decrease the risk of fractures, but do not improve bone quality. An alternative emerging concept of osteoporosis treatment aims to enhance bone formation by stimulating osteoblast differentiation^6–9^. The current anabolic treatments involve biological agents such as intermittent parathyroid hormone (PTH) and anti-sclerostin antibody. However, there are several concerns that include the risk of developing osteosarcoma due to prolonged use of teriparatide, a recombinant protein form of PTH^10^. In addition, immunogenicity due to humanized anti-sclerostin antibody, high costs in production and relative low stability are further concerns^11^. Therefore, new targets that would allow pharmacological-mediated induction of osteoblastogenesis are required to efficiently address the high frequency of bone loss in elderly population. Identification of such targets necessitates application of unbiased screening approaches that functionally assay the crucial targets important for early stages of osteoblast differentiation.

Assessing *in vitro* differentiation potential of calvarial osteoblast culture is one of the standard systems for studying the regulation of bone cell function^12^. A wide variety of approaches have been developed to study osteoblasts *in vitro*, including murine primary cell cultures, immortalized osteoblast-like cell lines, and human osteoblasts^12–16^. In combination, these methods have facilitated critical information on the regulation of osteoblast proliferation, differentiation, survival, and function. However, the current approaches to study osteoblast differentiation offer limited applicability for high-throughput screening (HTS) approaches. This is due to the use of immortalized cell lines^17^, application of lentiviral-based shRNA libraries^18^, and the use of reporter gene activity assays, which introduce artificial biases concerning readouts for differentiation^19^. Moreover, assaying osteoblast differentiation relies mainly on alkaline phosphatase (ALP) activity, which is quantified by a colorimetric readout, a low sensitivity method that cannot be combined with cellular analyses methodologies, such as immunofluorescence microscopy^20–23^.

Overcoming these limitations would require: (i) a physiologically relevant system that recapitulates the properties associated with osteoblast progenitor cells *in vivo*, (ii) the possibility for single gene perturbation, combined with (iii) a robust, cell-based quantitative readout for multiparametric analysis.

Here, we describe the implementation of a RNAi-based high-content screening (HCS) method with a functional genomics pipeline that allows to identify the crucial factor necessary for early osteoblast differentiation.

## Results

### Alkaline phosphatase activity determined by ELF 97 provides robust quantification of early osteoblast differentiation

**Figure 1:**
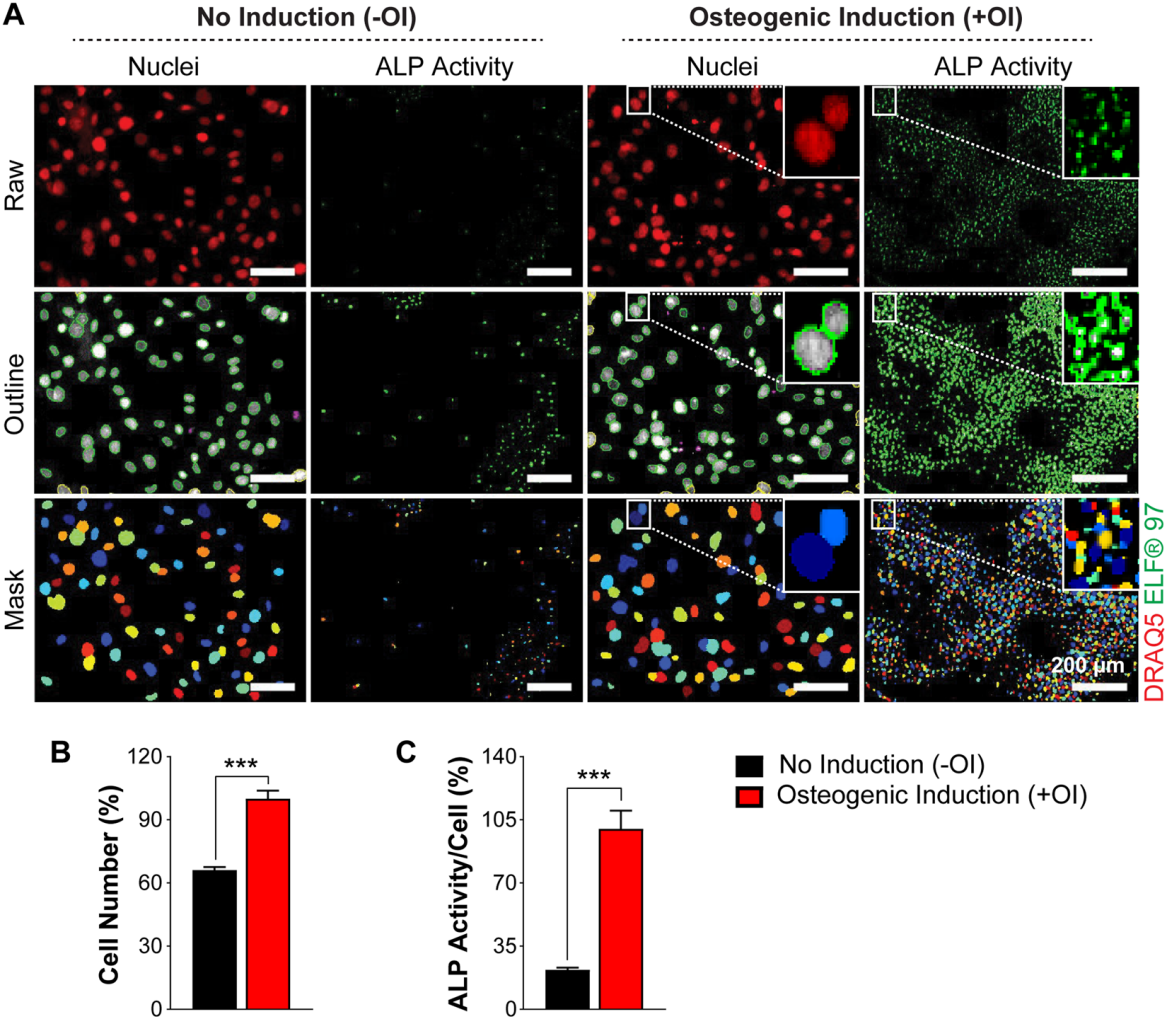
Representative images of undifferentiated (−OI) and differentiated (+OI) primary calvarial osteoblasts, and their cell identification and segmentation using CellProfiler. (**A**) The upper panel shows raw images of nuclei and alkaline phosphatase (ALP) staining of −OI and +OI condition in primary calvarial osteoblasts, stained with DRAQ5 (red) and ELF 97 (green) respectively. The middle panel depicts the outline of the nuclear and ALP staining of −OI and +OI treated primary calvarial osteoblasts. The lower panel shows the segmentation of nuclei and ALP by CellProfiler software from −OI and +OI exposed primary calvarial osteoblasts. The segmentation can be visualized by differences in colors between adjacent cells, in both nuclear and ALP channels (lower panel). (**B, C**) Percent cell numbers and cellular ALP activity in −OI and +OI conditions. Data are expressed as mean ± SEM (n = 6). Scale bar: 200 µm. *p < 0.05, **p < 0.01, ***p < 0.001.

First, we developed a sensitive, quantifiable readout for early osteoblast differentiation on a single cell level. In contrast to colorimetry, fluorescence-based methods are more sensitive, precise, specific, and can be utilized for multi-parametric analysis^20–23^. Therefore, we employed a fluorescence readout for quantitative analysis of alkaline phosphatase (ALP) activity using an established murine osteoblast-like cell line, MC3T3-E1 cells and primary calvarial osteoblasts derived from neonatal 129/Sv mice (primary osteoblasts). Both cell types exhibited increased ALP activity, upon osteogenic induction (+OI) compared to cells grown in normal media (−OI), as determined by conventional ALP staining (Supplementary Fig. S1A,B). Primary cells and MC3T3-E1 cells were able to generate mineralized nodules at day 20 of culture in response to osteogenic induction (+OI) as expected (Supplementary Fig. S2A–D). These results showed that both cellular systems were suitable to *in vitro* differentiation in our hands. To quantify the differentiation on cellular basis, we seeded both cell types in 384-well plates either under normal medium (−OI) or osteogenic induction medium (+OI) conditions. After six days of culture, the cells were fixed and stained with an ALP substrate called ELF97. This substrate is converted to bright and photostable yellow-green fluorescent precipitate at the site of enzyme activity. Using fluorescent microscopy, we imaged ELF 97 signal, and to avoid its spectral overlap with nuclear stains such as DAPI, we used DRAQ5 to stain the nuclei (Fig. 1A, upper panel). We then analyzed images from both channels using open-source, automated image analysis software, *CellProfiler*, to quantify the number of nuclei and the fluorescence intensity of the ELF 97 spots. In this software, we applied a channel dependent threshold for background reduction, along with object identification (Fig. 1A, middle panel) and segmentation (Fig. 1A, lower panel). The fluorescence intensity of ELF 97 spots was quantified and normalized to the number of cells represented by their nuclei within each image dataset to measure ALP activity on a per cell basis (Fig. 1B,C).

**Figure 2:**
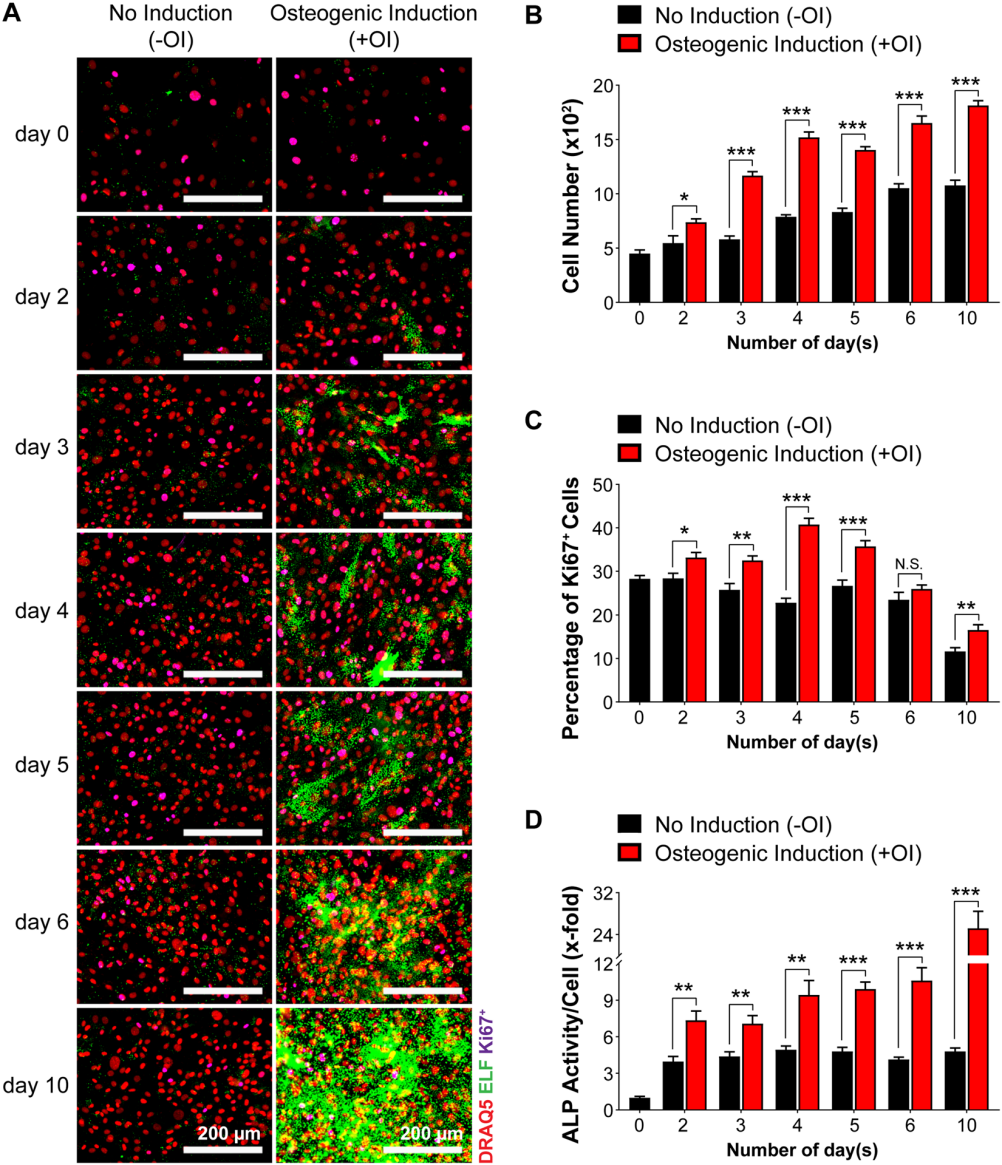
Quantification of alkaline phosphatase (ALP) activity and cellular proliferation during different stages of differentiation in primary calvarial osteoblasts.Primary calvarial osteoblasts were seeded in (**A**) 384-well plate and were grown up to 80% confluency. Subsequently, the cells were cultured either in the absence (−OI) or presence (+OI) of osteogenic induction medium for days indicated. (**A**) Cells were stained with DRAQ5 (red), Ki67 (purple), and ELF 97 (green) for nuclear, proliferative, and ALP staining respectively. (**B**) Quantification of cells shown in (**A**). (**C**) Percentage of Ki67^+^ cells (**D**) fold change in cellular ALP activity for −OI and +OI conditions at different time points. Data are expressed as mean ± SEM (n = 8). Scale bar: 200 µm. *p < 0.05, **p < 0.01, ***p < 0.001.

**Figure 3:**
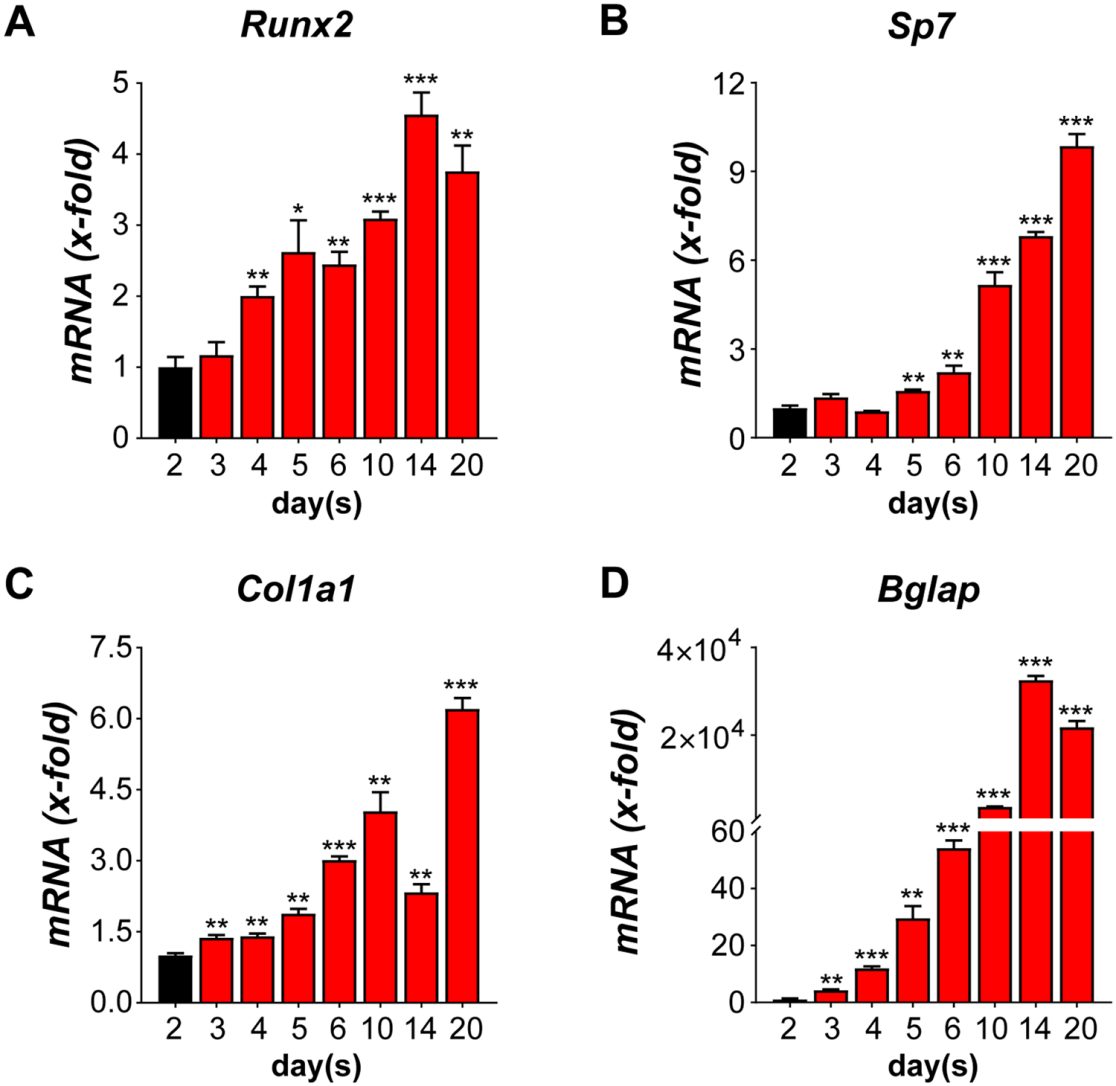
Expression of osteoblast-specific marker genes during osteoblast differentiation in primary calvarial osteoblasts. Expression of marker genes on specified days (**A**) Runx2, (**B**) Sp7, (**C**) Col1a1, and (**D**) Bglap. Data are expressed as mean ± SEM (n = 3). *p < 0.05, **p < 0.01, ***p < 0.001.

We applied this image analysis setup for evaluation of cellular ALP activity in MC3T3-E1 and primary calvarial osteoblasts. In MC3T3-E1 cells, nuclei were overlapping and were impossible to separate into individual cells using the automated software, giving a false cell number estimation (Supplementary Fig. S3). Thus, MC3T3-E1 cells are not suitable for this analysis due to their uncontrolled growth and formation of multiple layers. In contrast, primary osteoblasts grew as a monolayer, thus making it easier to identify and discriminate between individual cells (Fig. 1A, nuclei panels). Next, to determine the cellular ALP activity at different time points, we quantified ALP activity at different stages in primary calvarial osteoblasts. Upon osteogenic induction, the ELF 97 staining of primary osteoblasts gradually increased in a time-dependent manner compared to cells cultured in normal medium throughout the whole time-course (Fig. 2A,D). Importantly, our fluorescence-based method correlated well with the conventional ALP staining (Supplementary Fig. S1B). Intriguingly, upon osteogenic induction (+OI) we observed an increase in cell numbers of primary calvarial osteoblasts that correlated with increased Ki67 staining (Fig. 3A–C, Supplementary Fig. S4). The increased proliferation was accompanied with increased differentiation indicated by upregulation of runt related transcription factor 2 *(Runx2)*, Sp7 transcription factor 7 *(Sp7)*, collagen type I alpha 1 *(Col1a1)* and bone gamma carboxyglutamate protein *(Bglap)* throughout the time course (Fig. 3A–D). In line, ongoing increase of cell numbers has been observed in rodent cells during early phases of differentiation^24^. Overall, our data demonstrated that fluorescence-based ALP activity staining is useful for cell-based quantification of early differentiation of primary osteoblasts and can be utilized as one component for multi-parametric analysis.

### Primary calvarial osteoblasts can be efficiently transfected with siRNA without affecting cell number and differentiation

**Figure 4:**
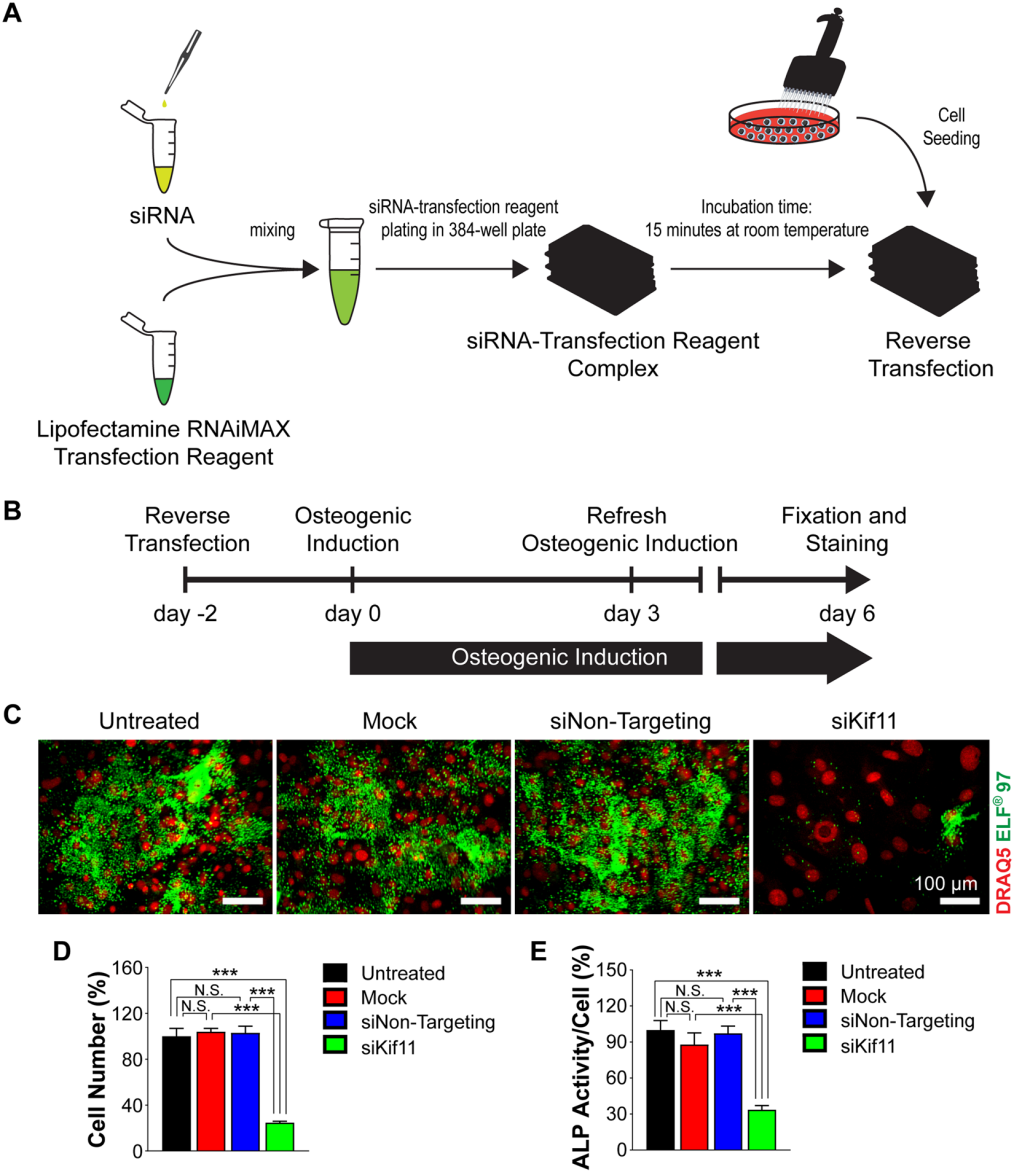
The efficiency of siRNA knockdown eight days after transfection in primary calvarial osteoblasts. (**A**) Scheme showing reverse transfection strategy. (**B**) Timeline showing the series of treatments during osteoblast differentiation in primary osteoblasts. (**C**) Representative microscopic images of primary osteoblasts showing DRAQ5 nuclear staining (red spheres) and ELF staining (green spots) after different treatments: untreated (media only), mock (transfection reagent only), siNon-Targeting (both siRNA and transfection reagent) and siKif11. (**D**,**E**) Percent cell numbers and cellular ALP activity after the treatments indicated in (**C**). Data are expressed as mean ± SEM (n = 6). Scale bar: 100 µm. *p < 0.05, **p < 0.01, ***p < 0.001.

We subsequently established a siRNA transfection procedure for primary calvarial osteoblasts in a 384-well format. Reverse transfection of siRNA complexed with the Lipofectamine RNAiMAX showed efficient transfection efficiency without hampering cell number and cellular ALP activity (Fig. 4A–E). We determined transfection efficiency indirectly by using siRNA against kinesin family member 11 (Kif11), a molecular motor protein essential for mitosis. The siRNA against Kif11 resulted in a cell cycle arrest for mitotic cells and exhibited large cell bodies leading to decreased cell number (Fig. 4C,D). Approximately 75% of cells were lost eight days after transfection indicating a high transfection efficiency in primary calvarial osteoblasts (Fig. 4C,D). In contrast, mock and Non-Targeting siRNA transfection did not affect cell numbers or ALP activity (Fig. 4C–E) indicating low cellular toxicity and allowing proper differentiation of primary calvarial osteoblasts. In addition to 384-well plate format, we further established delivery of siRNAs in primary calvarial osteoblasts for various plate formats as described in Supplementary Table S1.

### siRNA knockdown of known osteoblast regulators modulate ALP activity

**Figure 5:**
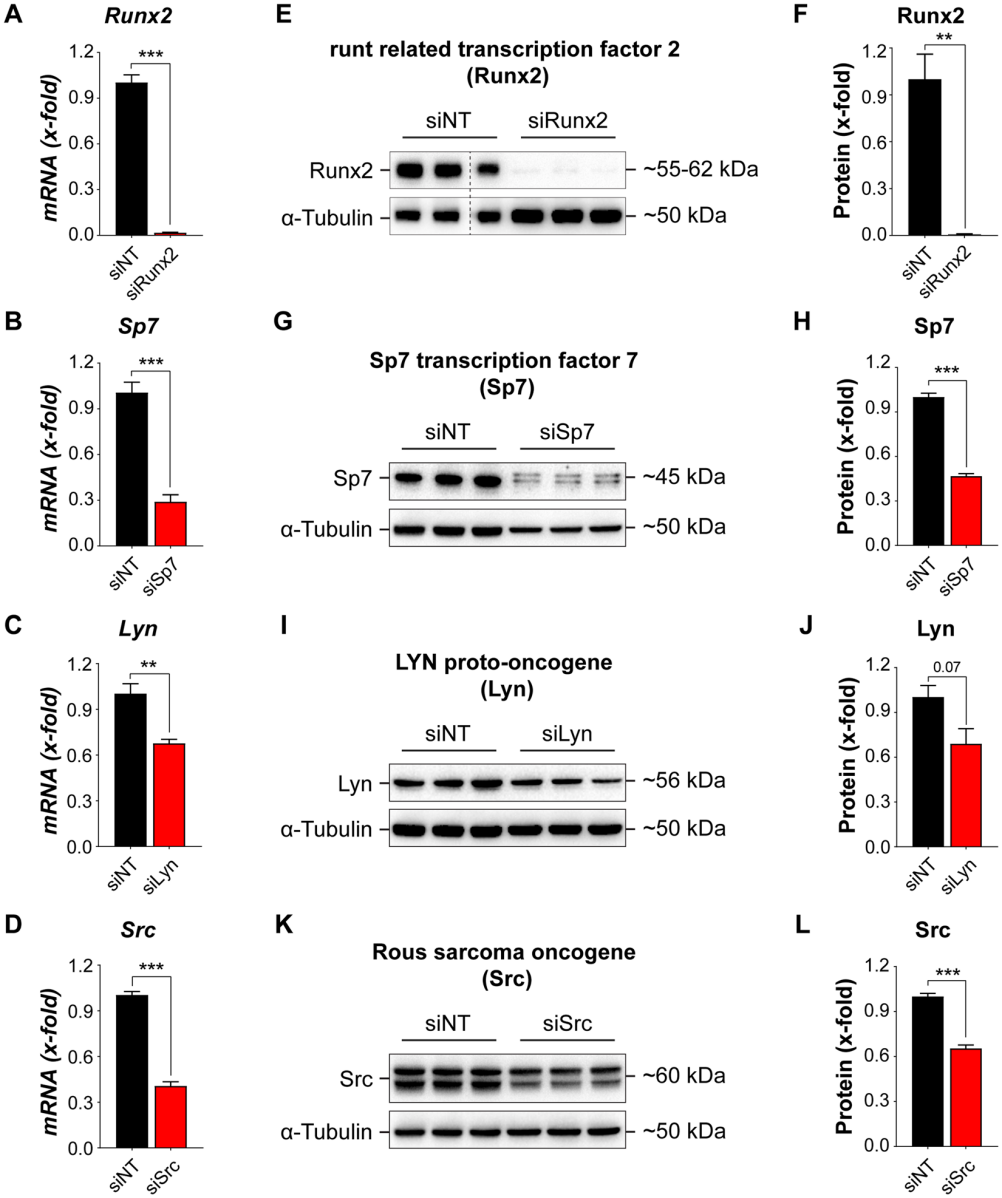
siRNA knockdown of known regulators of osteoblast differentiation in primary calvarial osteoblasts. Quantitative real-time PCR analysis of genes after siRNA knockdown for 8 days: (**A**) Runx2, (**B**) Sp7, (**C**) Lyn, and (**D**) Src. Western blot analysis of respective genes after siRNA knockdown for eight days: (**E**,**F**) Runx2 (the empty lane has been cropped, as shown with the dotted line, For original, see Supplementary Fig. S5). (**G**,**H**) Sp7, (**I**,**J**) Lyn, and (**K**,**L**) Src. Data are expressed as mean ± SEM (n = 3). *p < 0.05, **p < 0.01, ***p < 0.001.

**Figure 6:**
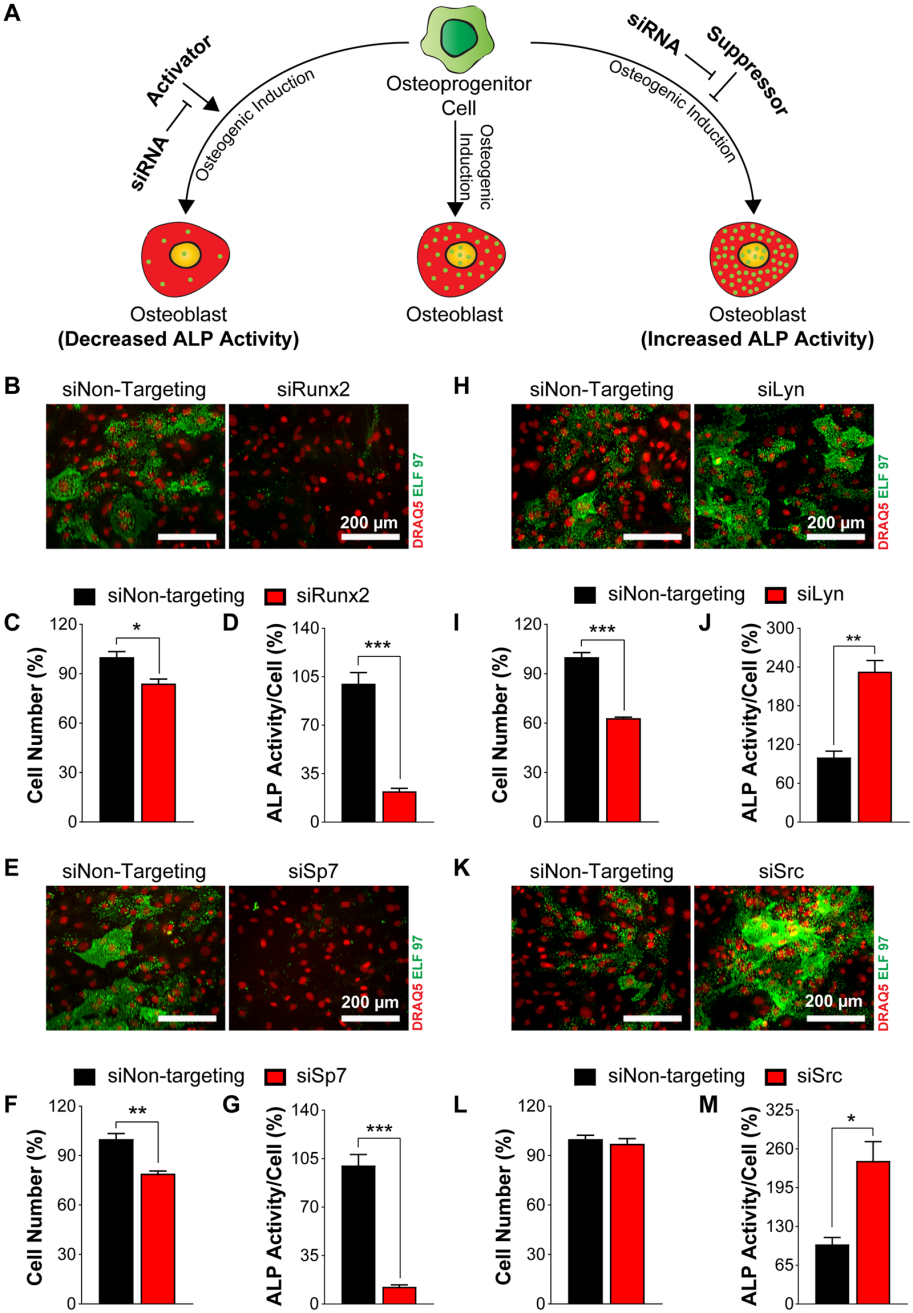
siRNA knockdown of known regulators modulates osteoblast differentiation in primary calvarial osteoblasts. (**A**) Scheme showing consequence of siRNA knockdown of regulators on osteoblast differentiation. (**B**,**E**) Representative microscopic images of DRAQ5 and ELF 97 stained cells after siRNA treatment against Sp7 and Runx2 using DRAQ5 (red) and ELF 97 (green) for nuclear and ALP staining, respectively. (**C**,**F**) Percentage of cell number after Runx2 and Sp7 siRNA knockdown, respectively. (**D**,**G**) Percent cellular ALP activity after Runx2 and Sp7 siRNA knockdown, respectively. (**H**,**K**) Representative microscopic images of DRAQ5 and ELF 97 stained cells after siRNA treatment against Lyn and Src, using DRAQ5 (red) and ELF 97 (green) for nuclear and ALP staining, respectively. (**I**,**L**) Percentage cell number after Lyn and Src siRNA knockdown respectively. (**J**,**M**) Percent cellular ALP activity after Lyn and Src siRNA knockdown respectively. Data are expressed as mean ± SEM (n = 3). Scale bar: 200 µM. *p < 0.05, **p < 0.01, ***p < 0.001.

To test whether siRNA knockdown of known activators and suppressors of osteoblast differentiation lead to changes in cellular ALP activity, we reverse transfected primary calvarial osteoblasts at day −2 with siRNA against known activators, [runt-related transcription factor 2 (Runx2) and Sp7 transcription factor 7 (Sp7)]^25–28^, and known suppressors, [LYN proto-oncogene (Lyn) and rous sarcoma oncogene (Src)]^29–33^. Analysis of mRNA (Fig. 5A−D) and protein (Fig. 5E–L, Supplementary Fig. S5) expression, at eight days post transfection demonstrates efficient knockdown. Furthermore, we reversed transfected the primary cells with siRNAs against Runx2, Sp7, Lyn, and Src in a 384-well format (Fig. 6). After 48 hours, we replaced the transfection medium with osteogenic induction medium and fixed the cells after six days of differentiation to assess cellular ALP activity (Figure 6A). There was a slight decrease in cell number upon siRNA knockdown of Sp7 and Runx2 as well as a drastic decrease in the cellular ALP activity, which corroborated well with previous studies (Fig. 6B−F). In contrast, knockdown of Lyn significantly reduced the cell number, but cellular ALP activity was significantly increased (Fig. 6H−J). Moreover, knockdown of Src did not influence cell number, but increased cellular ALP activity (Fig. 6K−M), confirming their suppressive role on differentiation.

### High-content screening of a siRNA library identified novel regulators of osteoblast differentiation

**Figure 7:**
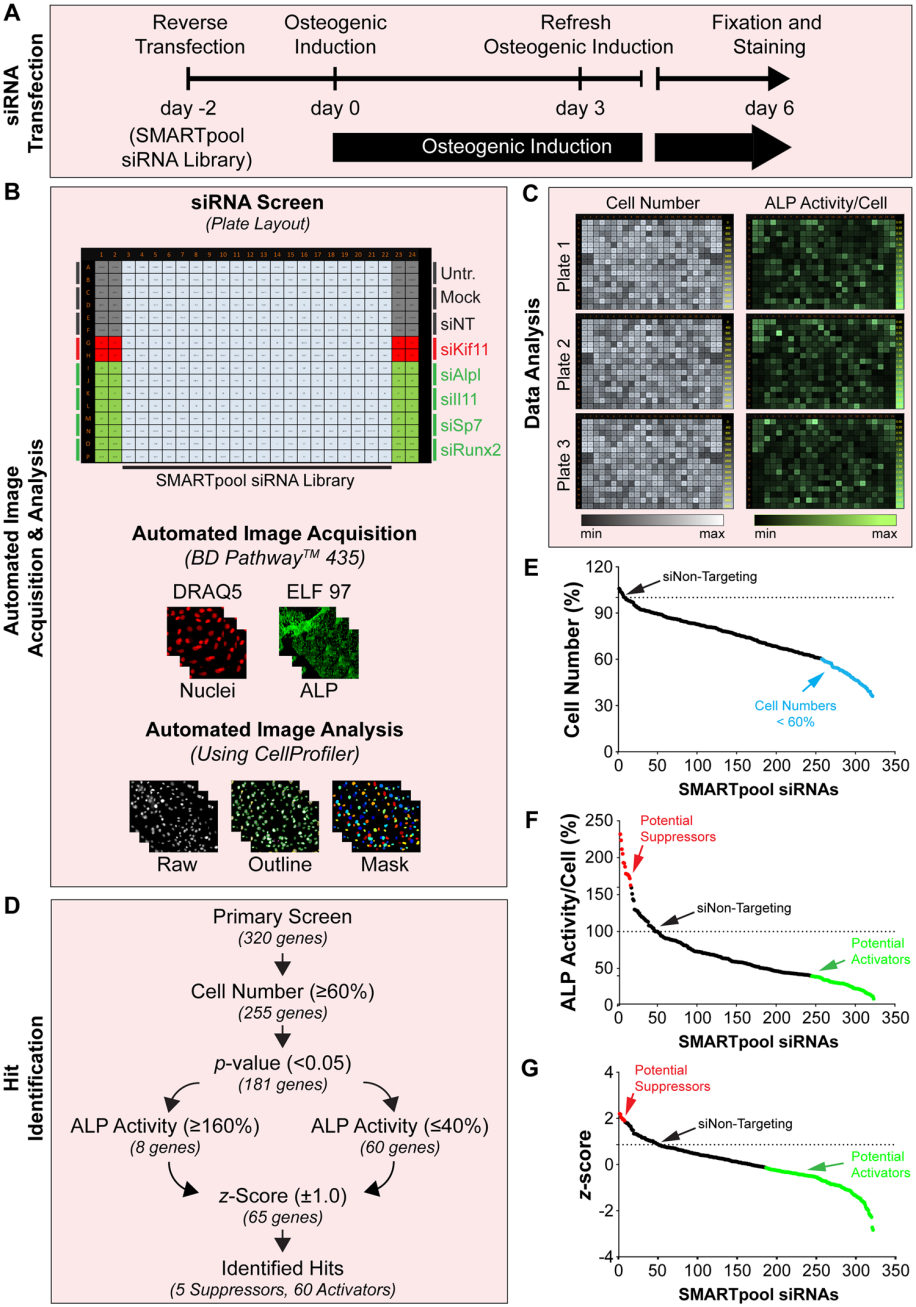
High-content analysis of a siRNA library plate to identify novel regulators of osteoblast differentiation.(**A**) Scheme showing reverse transfection and osteoblast differentiation strategy of SMARTpool siRNA library. (**B**) Plate layout for the siRNA screen and automated image acquisition and analysis setup. (**C**) Heatmap table for determining the reproducibility of the screening plate in triplicates. The heatmap table depicts cell numbers (minimum-maximum threshold: scale from 0–6000 cells, shown on right side of each plate) and ALP fluorescence intensity per cell (minimum-maximum threshold: scale from 0–3.75, shown on right side of each plate). (**D**) Scheme showing the criteria’s employed for hit identification. (**E**) Percentage of cell number of screened genes in the form of a rank plot (light blue dots represent siRNAs affecting cell number <60%). (**F**) Percentage of cellular ALP activity of siRNAs screened in the form of a rank plot (red dots represent siRNAs increasing ALP ≥160%, green dots represent siRNAs decreasing ALP ≤40%). (**G**) The z-Score ( ± 1.0 compared to Non-Targeting control) of screened genes in the form of a rank plot (red dots represent siRNAs with higher z-score ± 1.86, green dots represent siRNAs with lower z-score ± −0.14). The screening was done in triplicates.

After optimizing the conditions for HTS with known regulators of osteoblast differentiation (Runx2, Sp7, Lyn and Src) in 384-well format, we performed a siRNA screen against 320 genes in primary calvarial osteoblasts using SMARTpool siRNA library (Fig. 7A). We used siRNA targeting Kif11 as a transfection efficiency control whereas siRNAs targeting alkaline phosphatase (Alpl), interleukin 11 (Il11), Sp7 transcription factor 7 (Sp7) and runt related transcription factor 2 (Runx2) served as positive controls as known positive regulators of osteoblast differentiation^26,27,34–36^ (Fig. 7B, plate layout). We included Il11 as a well-known additional positive regulator of osteoblast differentiation^35,37,38^ (Supplementary Table S3). After fixation and staining, the fluorescent images were acquired with an automated BD pathway^TM^ 435 microscope, followed by the automated image analysis using CellProfiler (Fig. 7B, middle and lower panel). To determine the reproducibility of screening plate in triplicates, we generated a heatmap table of data for cell numbers and cellular ALP activity (Fig. 7C). The reduction of cell number upon siRNA against Kif11 (Fig. 7B for plate layout and 7 C), a transfection efficiency control, and a significant decrease in cellular ALP activity upon siRNA knockdown of the positive controls (Fig. 7B for plate layout and 7 C), served as quality control (Fig. 7B,C, Supplementary Table S3). For identification of positive hits, we firstly sorted the siRNA treatments based on their effect on cell number and displayed them in a rank plot (Fig. 7D,E). The siRNA treatments that reduced cell number less than 60%, were excluded from further analysis (Fig. 7D,E, Supplementary Table S3). Secondly, based on the cellular ALP activity of the positive controls, we set an arbitrary cut-off of ±60% ALP activity changes with respect to siNon-Targeting control (100%). Therefore, the siRNAs against genes that resulted in ≥160% or ≤40% ALP activity, as compared to the control with a *p*-value of <0.05, were considered as primary hits suitable for further validation (Fig. 7D,F, Supplementary Table S3). Lastly, we considered a z-Score of ±1.0 with respect to Non-Targeting control as a final criterion for hit selection. Therefore, the siRNAs that fall outside of ≥160% or ≤40% ALP activity, were considered as positive hits (Fig. 7D,G, Supplementary Table S3). Applying these criteria, we identified five potential suppressors (Table 1, Supplementary Table S3) and 60 potential activators (Table 2, Supplementary Table S3) of osteoblast differentiation. Interestingly, our screen was able to identify some known regulators for osteoblast differentiation, such as hepatocyte growth factor (Hgf)^39^, homocysteine-inducible endoplasmic reticulum stress-inducible ubiquitin-like domain member 1 (Herpud1)^40^, interferon activated gene 204 (Ifi204)^41^, and HPS3 biogenesis of lysosomal organelles complex 2 subunit 1 (Hps3)^42^. Additionally, we identified several genes with an unknown role in osteoblastogenesis, thus, opening new avenues of investigation in regulating osteoblast differentiation. Taken together, we here established a method to allow genome-wide high-content screening with siRNAs in primary calvarial osteoblasts to identify osteoblast genes necessary for early osteoblast differentiation.

**Table 1:** List of potential suppressors of osteoblast differentiation identified by RNAi screen.

Gene	ALP Activity/Cell (%)	*p*-value	Gene	ALP Activity/Cell (%)	*p*-value
H2-Ob	231.79	<0.05	H2-DMa	192.48	<0.01
Ifna9	205.22	<0.05	Il13ra2	187.73	<0.05
Icam5	193.19	<0.01

**Table 2:** List of potential activators of osteoblast differentiation identified by RNAi screen.

Gene	ALP Activity/Cell (%)	*p*-value	Gene	ALP Activity/Cell (%)	*p*-value
Hgfac	12.52	<0.001	Hrasls	29.82	<0.01
Ifna13	14.40	<0.001	Hat1	30.15	<0.01
Idi1	15.06	<0.001	Golgb1	30.26	<0.001
Icos	17.07	<0.001	Lilrb4	30.49	<0.001
Ssh1	18.38	<0.001	Hp	30.75	<0.001
Hmbs	19.25	<0.001	Hfe	30.79	<0.001
Ube2k	19.83	<0.001	Hcn2	30.92	<0.001
Igfbp1	20.25	<0.001	Herc1	31.25	<0.001
Glipr1	21.09	<0.001	Hrg	31.67	<0.001
Hspa5	21.38	<0.001	Hdac2	31.72	<0.001
Hip1	21.74	<0.001	H2-T23	31.89	<0.001
Ifi30	22.19	<0.001	Hoxc4	31.92	<0.001
Herpud1	22.43	<0.001	Icam1	33.13	<0.001
Herc2	22.60	<0.001	Hpcal1	33.40	<0.001
Golga5	22.68	<0.001	Ifi204	34.07	<0.01
Hdac7	22.85	<0.001	Hivep2	34.29	<0.01
Gnmt	22.92	<0.001	Ifnar1	34.37	<0.001
Hal	23.37	<0.001	Hps3	35.06	<0.001
Hmga2	23.53	<0.001	Ifit2	36.49	<0.001
Hcrtr1	25.64	<0.001	Hsf2	37.11	<0.01
Hey2	26.08	<0.001	Hdac3	37.29	<0.001
Hmgn2	27.29	<0.001	Ifna4	38.04	<0.001
Hspb1	27.62	<0.001	Hoxd12	38.15	<0.01
Sap30bp	28.38	<0.001	Gpr135	38.43	<0.001
Hrc	28.65	<0.01	Hgf	38.54	<0.001
Ifngr2	29.00	<0.001	Hdac11	38.70	<0.001
Hus1	29.61	<0.001	Hsd11b2	38.72	<0.01
Hoxb5	29.71	<0.001	Glo1	38.83	<0.001
Ifit3	29.78	<0.001	Hnf4g	39.32	<0.001
Il11	29.79	<0.001	Hesx1	39.62	<0.01

## Discussion

Understanding the molecular mechanism of osteoblast differentiation and bone-related disorders is a challenge in bone biology^43^. Thus, several approaches have been employed including HTS of small molecule inhibitors to identify regulators that promote osteoblast differentiation in C2C12 or MC3T3 cell lines^17^. However, there are certain limitations associated with these approaches, which include the use of osteoblast-like cell lines, less sensitive readout to determine ALP activity, and the lack of cell-based multiparametric analysis^17,44^. In this study, we overcome these caveats by developing a fluorescent microscopy-based method in physiologically relevant primary calvarial osteoblasts to determine the cellular ALP activity. This method can be used to precisely quantify the cell number and proliferation, to determine cellular ALP activity, and can be utilized for multiparametric analysis.

Other strategies that have been used so far include the lentiviral-based shRNA screens to find out novel osteoblast differentiation regulators^18^ and the use of immortalized cell lines in combination with reporter genes under the control of osteoblast specific promoters as a readout for osteoblast differentiation^19^. Nevertheless, there are several drawbacks associated with these approaches, which include the use of lentiviral-based shRNA system, which is time-consuming, expensive, and requires high safety measures^45–47^. In addition, the use of luciferase assay as an artificial readout reflects the regulation of one gene and not the entire cellular phenotype for differentiation^19^. In contrast, we present a high-content siRNA screening method with multiple readouts for cell numbers, proliferation, and differentiation to identify regulators of osteoblastogenesis in primary calvarial osteoblasts. This method is relatively fast, easy to handle, and does not require higher level safety measures. Though the effect of siRNA is transient, it can be utilized to identify factors responsible for early stages of osteoblast differentiation.

Although primary cells are difficult to transfect^48^, we successfully established an efficient siRNA transfection method for primary calvarial osteoblasts. The siRNA treatment caused a significant decrease in mRNA and protein expression of Sp7, Runx2, Lyn, and Src, respectively. Accordingly, we found a significant decrease or increase in cellular ALP activity upon siRNA knockdown of these known activators, Sp7 and Runx2^25–28^, or suppressors, Lyn and Src^29–33^, respectively. Intriguingly, siRNA knockdown of Lyn resulted in a significant decrease in cell number, which could lead to misinterpretation of data during a bulk analysis based on a total readout from a well. Our method circumvents this issue as it allows quantification of ALP activity on a single cell basis enabling accurate quantification and eliminating the bias resulting from altered cell numbers in a scenario similar to Lyn siRNA knockdown.

Here, we demonstrate that the application of this screening method for siRNAs against 320 genes lead to the identification of five potential suppressors and 60 activators. Interestingly, some of the identified genes are well-known regulators of osteoblast differentiation such as Hgf that promotes osteoblast differentiation through p38 signaling pathway^39^ and Herpud1, which enhances osteoblast maturation and mineralization^40^. These findings therefore confirm the validity of the procedure. We also identified several genes with unspecified role in osteoblastogenesis, thus, opening new avenues of investigation in regulating osteoblast differentiation. Moreover, this method is not restricted to RNAi-based screening but can be readily adapted to large-scale drug HTS or to small-scale targeted experiments.

In conclusion, we successfully established a fluorescent microscopy-based method for high-content RNAi screening in primary calvarial osteoblasts and identified potential modulators crucial for early osteoblast differentiation.

## Materials and Methods

### Isolation of primary calvarial osteoblasts

Primary calvarial osteoblasts were isolated from 129/Sv neonatal mouse calvaria of 3–5 days after birth, as previously described^13^. Briefly, after isolation, the calvariae were put into 1 ml digestion solution (0.1% w/v of each Collagenase A (Cat. 11088793001, Roche) and Dispase II (Cat. 04942078001, Roche) at 37 °C while shaking at 700 rpm. The first supernatant was discarded (fraction 1). The digestion was repeated four more times (fraction 2 to faction 5), and the supernatant was collected in 15 ml falcons containing 500 µl of fetal bovine serum (FBS) (Cat. A15–101, GE Healthcare). The samples were kept on ice during the whole procedure. The supernatant collected was centrifuged at 1500 rpm for 5 minutes at RT. The cell pellet was resuspended in complete culture medium (α-MEM (Cat. 41061037, GIBCO) supplemented with 10% FBS (Cat. A15.101, GE Healthcare) and 1% penicillin/streptomycin (Cat. P0781, Sigma-Aldrich). The cell suspension was plated in 6-well plates (using one well per calvaria) and was kept in 5% CO_2_ incubator at 37 °C overnight. Next day, the cells were washed with 1x PBS to remove any non-adherent cells and the media was replaced with fresh complete medium. The cells were allowed to grow for 2–3 days until they reached approximately 80% confluency. The cells from each 6-well plate were collected by trypsinization, pooled, and plated on the 10 cm dishes. Upon reaching 80% confluency, the cells were then used for the experiments.

### Cell culture

Primary osteoblasts were maintained in complete medium (α-MEM (Cat. 41061037, GIBCO) supplemented with 10% FBS (Cat. A15.101, GE Healthcare) and 1% penicillin/streptomycin (Cat. P0781, Sigma-Aldrich). MC3T3-E1 osteoblastic cell line (ATCC) was cultured in complete medium (α-MEM (Cat. A1049001-01, GIBCO) supplemented with 10% FBS (Cat. A15.101, GE Healthcare) and 1% penicillin/streptomycin (Cat. P0781, Sigma-Aldrich).

### siRNAs

The SMARTpool siRNAs targeting mouse Kif11 (Cat. M-040880-00-0005), Runx2 (Cat. M-064819-02-0005), Sp7 (Cat. M-045886-01-0005), Non-Targeting # 2 (Cat. D-001206-13-05), Il11 (Cat. M-046578-01-0005), Alpl (Cat. M-043406-01-0005), Lyn (Cat. M-040987-01-0005), Src (Cat. M-040877-01-0005) and SMARTpool siRNAs mouse libraries (Cat. G-013500, G-013600, G-014600) were all purchased from Dharmacon. (ThermoFisher Scientific).

### siRNA transfection

The SMARTpool siRNAs arrived as lyophilized powder and were stored at −20 °C. For siRNA transfection in a 384-well plate, a 500 nM master stock of the resuspended library was used (Supplementary Table S1)^21^. All positive controls (Alpl, Il11, Sp7 and Runx2) were also diluted to the same concentration. For each well, 3.2 µl of this stock was pipetted into an intermediate 384-well plate (Cat. 781281, Greiner). Another 16.8 µl of Opti-MEM (Cat. 31985047, Life Technologies) containing 0.096 µl of Lipofectamine RNAiMAX (Cat. 13778-150, Life Technologies) was added. From the intermediate plate, 20 µl were then transferred to each well of the screening plate (Cat. 353962, BD/Corning/Falcon) *(Note: experiments were done in triplicates)*. The procedure was either performed with a TECAN pipetting workstation (Tecan Freedom EVO with MCA96) or multichannel pipette. The siRNA-transfection reagent complex was incubated for approximately 15 minutes at RT, followed by addition of 60 µl of cell suspension containing 1800 cells per well using BioTek cell dispenser. The final concentration of siRNA was 20 nM and that of Lipofectamine was 0.12% (Supplementary Table S1). The plates were then placed in the incubator (37 °C, 5% CO_2_) to allow the cells to adhere to the surface of the plate and siRNAs to enter into the cells. However, for siRNA transfection in larger wells/dishes, a 20 µM master stock was prepared using 1x siRNA buffer and a final concentration of 20 nM was used (Supplementary Table S1).

### Osteogenic induction

After 48 hours of cell seeding or transfection, the media was replaced with osteogenic induction medium (α-MEM (Cat. 41061037, GIBCO) supplemented with 10% FBS (Cat. A15.101, GE Healthcare) and 1% penicillin/streptomycin (Cat. P0781, Sigma-Aldrich), 100 µg/ml ( + )-Sodium L-Ascorbate (Cat. A4034, Sigma-Aldrich) and 5 mM β-glycerophosphate (Cat. G9422, Sigma-Aldrich). For 384-well plates, initially the media was discarded by spilling of media from plates into an autoclaved plastic box with its bottom covered by autoclaved paper. To get rid of the remaining media, the plates were gently tapped onto a new autoclaved paper inside the hood. Any formation of bubbles or foam within the wells while discarding media, should be avoided, which may hinder dispensing of new media (*Note: in case of foam or bubble formation, spray 70% ethanol on a new autoclaved tissue, tap and wipe the plate gently in an inverted position*). The 80 µl of freshly prepared osteogenic induction medium were then added using BioTek MicroFlo Select Dispenser (Fisher Scientific). The osteogenic induction medium was replaced after every third day.

### Cell fixation and ELF 97 phosphatase staining

For the 384-well plates, a pipetting workstation (Tecan Freedom EVO with MCA96) was used for fixation, washing, and DRAQ5 and ELF 97 staining. The workstation contains a washer module (TECAN Powerwasher PW384 or HydroSpeed), which was applied for all washing steps. After eight days of siRNA transfection, the plates were washed once with 1x PBS and the buffer was removed using the washer. Next, 40 µl of 4% paraformaldehyde was added to each 384-well and incubated for 10 minutes at RT. The cells were then washed three times (5 minutes each) by adding 80 µl of 1x PBS to each 384-well. The procedure was followed by cell labelling. Firstly, the cells were permeabilized using 30 µl 0.2% Tween 20 (Cat. 9127.2, Carl Roth) in 1x PBS and incubated for 10 minutes at RT. The plates were washed three times with 1x PBS (volume = 80 µl) using the washer. Next, the ELF 97 phosphatase substrate (Component A), and detection buffer (Component B) was mixed in a ratio of 1:20 to prepare the ALP detection solution (Cat. E6601, Thermo Fischer Scientific) (10 µl for each well) (*Note: prepare the substrate solution in the dark and only the required volume for the day’s experiment*). To each well, 10 µl of ALP detection solution was added and incubated for 5 minutes at RT *(Note: perform the whole procedure in the dark)*. The cells were washed again three times with 1x PBS (volume = 80 µl) using the washer to stop the ALP detection reaction. Next, the buffer was removed and 20 µl of the nuclear stain DRAQ5 (1:1000 of stock solution in 1x PBS) (cat. DR51000, BioStatus) was added and incubated for 30 minutes at RT. The plates were washed again three times with the washer. Finally, the washing buffer was removed and 80 µl of 1x PBS containing 0.1% v/w sodium azide was added per well. The plates were covered with plate sealer (Cat. 676070, Greiner Bio-One) and stored at RT overnight in the dark to reduce non-specific cytoplasmic DRAQ5 staining. Next day, the plates were subjected to imaging using the automated fluorescence microscope (BD pathway 435).

### Cell proliferation assay

For the measurement of cell proliferation, the cells were stained with Ki67 antibody. The staining was done after the measurement of ELF 97 to avoid reduction of the ELF 97 signal due to the use of a stronger permeabilization agent (Triton X-100) and further washing steps. Therefore, after imaging ELF 97 and DRAQ5, the plates were washed again with 1x PBS (volume = 80 µl) using washer. Next, the cells were permeabilized with 0.2% Triton X-100 (Cat. 3051.3, Carl Roth) in 1x PBS and incubated for 20 minutes at RT. The cells were washed again three times with 1x PBS and 30 µl of immunofluorescence (IF) blocking buffer^25^ was added and incubated for 30 minutes. The buffer was removed and 20 µl of Ki67 primary antibody (1:100 in IF blocking buffer, #MA5-14520, Thermo Pierce) was pipetted to each well and incubated for 45 minutes at RT. The cells were washed again four times with 1x PBS using washer. The buffer was then removed and 20 µl of secondary antibody Alexa Flour 488 (1:400 in IF blocking buffer, # A11034, ThermoFisher Scientific) was added and incubated for 30 minutes at RT. Afterwards, the cells were gently washed six times with 1x PBS using washer. Lastly, the washing buffer was removed and 80 µl 1x PBS containing 0.1% v/w sodium azide was added to each well.

### Image acquisition

The fluorescent images were acquired with an automated BD pathway 435 (BD Biosciences) fluorescence microscope using 10x objective and a xenon lamp. Automatic image acquisition mode with hardware autofocus was used for all the experiments^21^. For experiments with higher plate number, a robotic plate handler (Twister II) was used to automatically load plates on the microscope. Each well of the 384-well plates was imaged as an image set of two channels with 15 fields covering approximately 80% of the well area with a binning of 2. The nuclear staining with DRAQ5 was measured with an excitation filter of 628/40 nm, a dichroic filter of 660 nm, and an emission filter of 692/40 nm. The ALP activity with ELF 97 staining was measured with an excitation filter of 377/50 nm, a dichroic filter of 409 nm, and long pass emission filter of 435 nm. For the timeline experiment (DRAQ5, Ki67 and ELF staining) the image acquisition has been done using ImageXpressMicro Confocal (Molecular Devices) with a 10x objective, LED-illumination, and a high-resolution Scientific CMOS camera. Each well was measured with four fields covering 40% of the well in a set of 2 images for ELF and DRAQ5 or an additional one for Ki67 measurement. Here, the nuclear staining with DRAQ5 was measured with an excitation filter of 640/20 nm, a dichroic filter of 660 nm, and an emission filter of 692/40 nm. The ALP activity with ELF 97 staining was measured with an excitation filter of 395/50 nm, a dichroic filter of 409 nm, and emission filter of 536/40 nm. The Ki67 staining using Alexa Flour 488 was measured with an excitation filter of 480 nm, a dichroic filter of 506 nm, and an emission filter of 536/40 nm.

### Image analysis

Individual cells (stained with DRAQ5) or ALP activity (stained with ELF 97) from each image dataset were identified, segmented, and quantified using free online software CellProfiler^21,49^. The two channels were loaded into CellProfiler as “Nuclear” and “ELF” channels. In the nuclear channel, an adaptive thresholding method “otsu” was used for identification and masking of nuclei. Before the identification of the ALP activity foci, a background correction of the ELF-channel was applied using a median filter (object size = 10, smoothing filter size = 20). Next, an automated background global thresholding was applied (object size = 10, smoothing filter size = 20) for identification. The number of nuclei and the ELF 97 intensity within the ELF 97 spot area were quantified. For timeline experiment, the images from ImageXpressMicro Confocal were analyzed with the MetaXpress (MD) software using a top hat filter for background correction (feature size = 50 for DRAQ5 and 100 for ELF 97 and Ki67) of all channels. The nuclei were identified with the round object detection (size limit: 4-21.22 µm, threshold: 200 a.u.). The ELF 97 spots were identified applying a top hat filter of 10, followed by a round object detection (size limit: 1.3–8 µm, threshold: 1000 a.u.). The average ELF intensity of the background corrected ELF image of each set was measure within the area of the detected spots. This was then related to the number of nuclei within this area. For Ki67 positive cells, the average intensity of each nucleus was calculated and those which had an average intensity over a certain threshold (300 a.u.) were considered as Ki67 positive cells.

### Data analysis

The results of the image quantification generated by the CellProfiler software were imported into an excel spreadsheet. For each well, the number of cells per well was calculated as the sum of the detected nuclei of all 15 measured fields. The ALP activity was determined by the ELF signal intensity within spot area per field divided by the number of nuclei within this field and averaged between the fields of one well. The results of the ALP activity and the cell number were normalized as a percentage of the corresponding average of the Non-Targeting controls. For normalization of the ALP-activity per plate also the z-score of the binary logarithmized values was used as previously described^50^. For hit identification within triplicates, multiple t-testing of the binary logarithmized values of a sample against the Non-Targeting control was applied.

### Quantitative real-time PCR

Total RNA from primary calvarial osteoblasts was prepared using RNeasy Midi kit (Cat. 75142, Qiagen) according to the manufacturer’s instructions. The cDNA was synthesized using RevertAid H Minus Reverse Transcriptase (Cat. EP0451, ThermoFisher Scientific). The cDNA samples were then analyzed by ABI ViiA-7 Real-Time PCR system (ThermoFisher Scientific). The primer sequences used for real-time PCR are shown in Supplementary Table S2.

### Protein isolation and quantification

Cells were harvested in 1x PBS and centrifuged at 15,000 rpm for 5 minutes at 4 °C. The supernatant was discarded and the pellet was dissolved in RIPA lysis buffer (50 mM Tris-Cl (pH 8.0), 150 mM NaCl, 1% NP-40, 0.1% SDS, and 0.5% deoxycholate) supplemented with protease and phosphatase inhibitors (Complete Mini: Cat. ROC 11836153001, Roche; 1 mM sodium orthovandate, 1 mM phenylmethylsulfonylfluoride, 25 mM sodium fluoride, Sigma). The samples were shortly vortexed and kept on shaker at 700 rpm for 30 minutes at 4 °C. Cell debris was removed by centrifuging the samples at 15,000 rpm for 5 minutes at 4 °C. The supernatant was collected in a fresh tube and the protein concentration was determined by using the Pierce BCA protein assay kit (Cat. 23225, ThermoFisher Scientific) by following the manufacturer’s instructions.

### Western blotting

Western blotting analysis was performed as previously described^51^. Briefly, 30 µg of protein was diluted in 5x Laemmli buffer and then boiled at 95 °C for 5 minutes. The proteins were separated by SDS-PAGE and blotted on to a nitrocellulose membranes. The membranes were then blocked with 5% non-fat dry milk in 1x TBST for 30 minutes. Next, the membranes were incubated with primary antibodies against Runx2 (1:100, #12556, Cell Signaling Technology), Sp7 (1:500, ab22552, abcam), Lyn (1:200, #2732, Cell Signaling Technology) Src (1:200, #2109, Cell Signaling Technology) and α-Tubulin (1:100, Cat. T9026, Sigma-Aldrich), diluted in 5% non-fat dry milk at 4 °C overnight. Next day, membranes were washed three times with TBST for 5 minutes at RT, followed by the incubation with secondary antibodies (Anti-rabbit: 1:5000, #65–6120, Invitrogen and Anti-Mouse: 1:5000, Cat. P0447, Dako), diluted in 5% non-fat dry milk at RT for 1 hour. Before addition of chemiluminescence substrate, the membranes were washed three times and the desired protein band was detected using ChemiDoc MP imaging system (BIO-RAD). Quantitative analysis of the proteins was performed using ImageJ software. The densitometry values were normalized to the loading control α-Tubulin.

### Alkaline phosphatase staining

For qualitative ALP staining, MC3T3-E1 osteoblastic cell line (ATCC) and primary osteoblasts were seeded at 5,000 and 12,500 cells/cm^2^ in 24-well plates, respectively. The cells were grown until they reached 80% confluency. The complete culture medium was replaced with osteogenic induction medium (α-MEM (Cat. 41061037, GIBCO) supplemented with 10% FBS (Cat. A15.101, GE Healthcare) and 1% penicillin/streptomycin (Cat. P0781, Sigma-Aldrich), 100 µg/ml ( + )-Sodium L-Ascorbate (Cat. A4034, Sigma-Aldrich) and 5 mM β-glycerophosphate (Cat. G9422, Sigma-Aldrich)) and was changed after every third day. The MC3T3-E1 and primary osteoblasts were fixed with 4% paraformaldehyde at different time-points. ALP staining was done by using ALP kit (Fast Violet B Salt: Cat. 851, Sigma-Aldrich and Naphthol AS-MX phosphate: Cat: 855, Sigma-Aldrich) by following manufacturer’s protocol. Approximately 200 µl of solution was added to each 24-well and stored in dark for 1 hr at RT and washed with autoclaved water. The plates were then subjected to stereomicroscopy (Cat. M125 C, Leica Microsystems).

### Alizarin Red S staining and quantification

For qualitative staining, mineralization of MC3T3-E1 and primary osteoblasts was determined by staining the nodules with 1% w/v Alizarin Red S (Cat. A5533, Sigma-Aldrich) in a 24-well plate as previously described^52^. Briefly, the cells were fixed with 4% paraformaldehyde (Cat. 0335.3, Carl Roth) for 10 minutes at RT. The cells were washed three times with autoclaved water. Approximately 250 µl of 1% w/v Alizarin Red S stain was added to the each well and incubated in dark and periodically monitored for the staining to avoid the over staining. After staining, the cells were again washed three times with water. The liquid was removed and the plates were subjected to stereomicroscopy (Cat. M125 C, Leica Microsystems). For the quantitative staining, the Alizarin Red S staining was quantified as previously described^53^. Briefly, 200 µl of 10% acetic acid (Cat. 20104.334, VWR Chemicals) were added to each well of a 24-well plate and incubated at RT for 30 minutes while shaking at 700 rpm. With the help of cell scrapper, the cells were gently scrapped from the plate and transferred along with acetic acid into 1.5 ml Eppendorf tube. The tubes were vigorously vortexed for approximately 30 seconds, heated at 85 °C for 10 minutes and then transferred to ice for 5 minutes to cool down the samples. Next, the slurry was centrifuged at 15,000 rpm for 15 minutes at RT. In the meantime, the Alizarin Red S standards were prepared. After the centrifugation was finished, 200 µl of supernatant was transferred to new 1.5 ml tubes. For quantification, 100 µl of this supernatant was added to a transparent bottom 96-well plate along with 100 µl of standards. Finally, to neutralize the pH, approximately 100 µl of 10% ammonium hydroxide was added. The plate was read at OD_405_.

### DNA content

The DNA content was determined as previously described^54^. Briefly, 2 µl of DNA was added to a corning clear bottom 96-well black microplate with 48 µl of assay buffer. The procedure was followed by the addition of 50 µl of fluorescent dye bisBenzimide H 33258 (Cat. Hoechst 33258, Sigma-Aldrich). A 1 µg/ml solution of dye was used. The plate was incubated for 10 minutes in a dark, followed by measurement of fluorescence using CLARIOstar (BMG LABTECH) 96-well plate reader (bottom optic, excitation wavelength ± bandwidth: 360 ± 20 nm, emission wavelength: 460 ± 30 nm). The DNA content was quantified by using the Calf Thymus DNA as standard.

### Statistical analysis

The results are expressed as mean ± standard error mean (SEM). Differences between the groups were determined by unpaired homoscedastic two-tailed student’s *t*-test. The *p*-value less than 0.05 was regarded as a statistically significant difference, *p < 0.05, **p < 0.01, ***p < 0.001.

## Electronic supplementary material

Supplementary Information.

Supplementary Table S3
