# Supplementary material for: Cell-based RNAi screening and high-content analysis in primary calvarian osteoblasts applied to identification of osteoblast differentiation regulators
Source: Sci Rep. 2018 Sep 19;8:14045. doi: 10.1038/s41598-018-32364-8 (PMC6145911; doi:10.1038/s41598-018-32364-8)

A cell-based RNAi screening procedure and high-content analysis to identify novel regulators of osteoblast differentiation in primary calvarial osteoblasts.

Mubashir Ahmad^1^, Torsten Kroll^2^, Jeanette Jakob^2^, Alexander Rauch^2^, Aspasia Ploubidou^2^, Jan Tuckermann^1, 2^

^1^Institute for Comparative Molecular Endocrinology (CME), Ulm University, Helmholtzstrasse 8/1, 89081 Ulm, Germany.

^2^Leibniz Institute on Aging – Fritz Lipmann Institute (FLI), Beutenbergstrasse 11, D-07745 Jena, Germany.

Author for Correspondence: [jan.tuckermann@uni-ulm.de](mailto:jan.tuckermann@uni-ulm.de), Tel.: +49 (0)731/50-32600, Fax: +49 (0)731/50-32609.

# Supplementary Information

**Supplementary Figure S1**


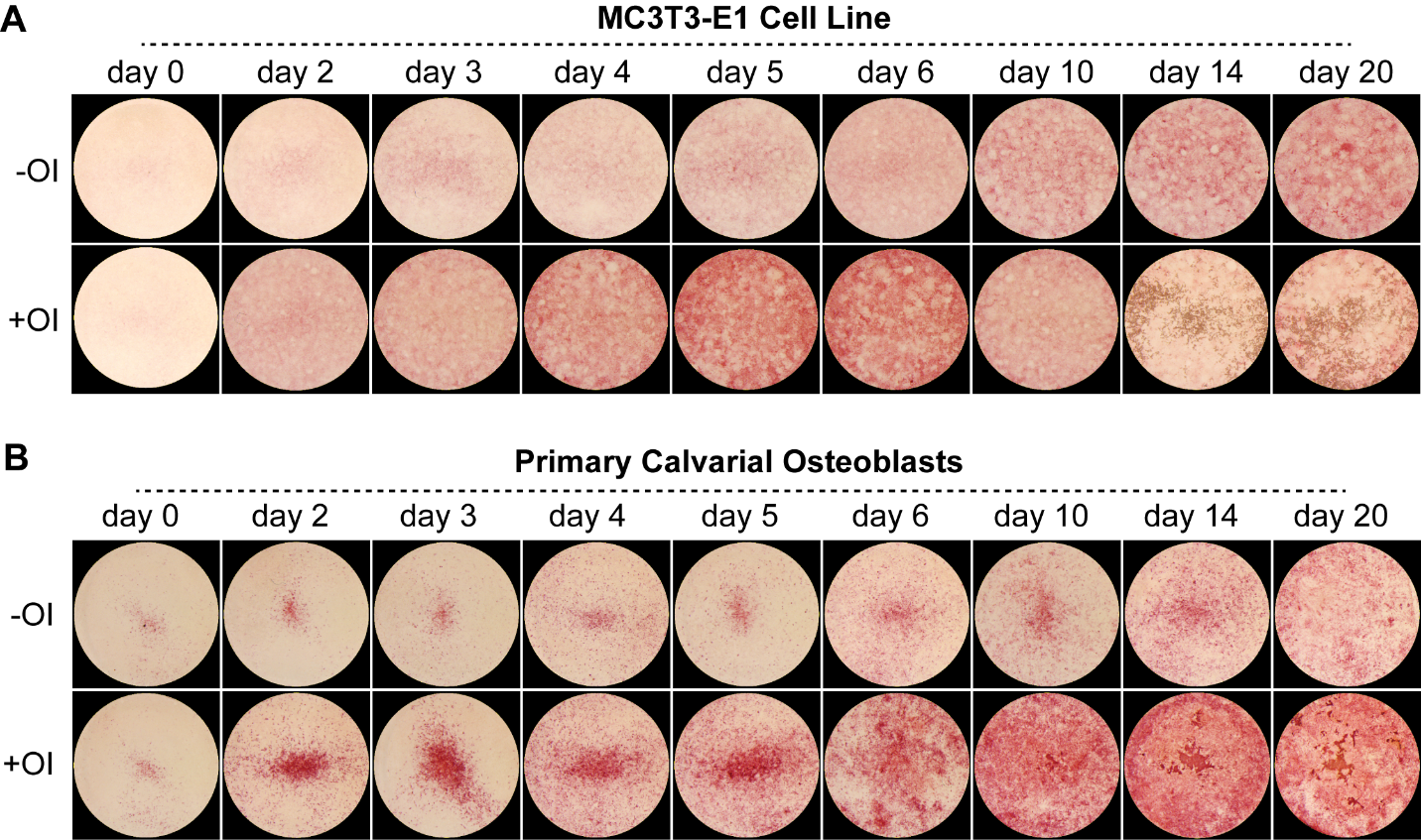


Supplementary Figure S1. Differentiation pattern observed through alkaline phosphatase (ALP) staining in MC3T3-E1 osteoblastic cell line and primary calvarial osteoblasts.

(A, B) Representative images of alkaline phosphatase (ALP) staining of MC3T3-E1 (n = 2) and pO-129/Sv (n = 2) at day 0, 2, 3, 4, 5, 6, 10, 14, and 20. MC3T3-E1 cells and primary osteoblasts were seeded in 24-well plates. At 80% confluency, the cells were cultured either in the absence (-OI) or presence (+OI) of osteogenic induction medium until fixed on the specified days. The ALP activity was monitored using conventional qualitative ALP staining method. The experiment was repeated three times.

**Supplementary Figure S2**


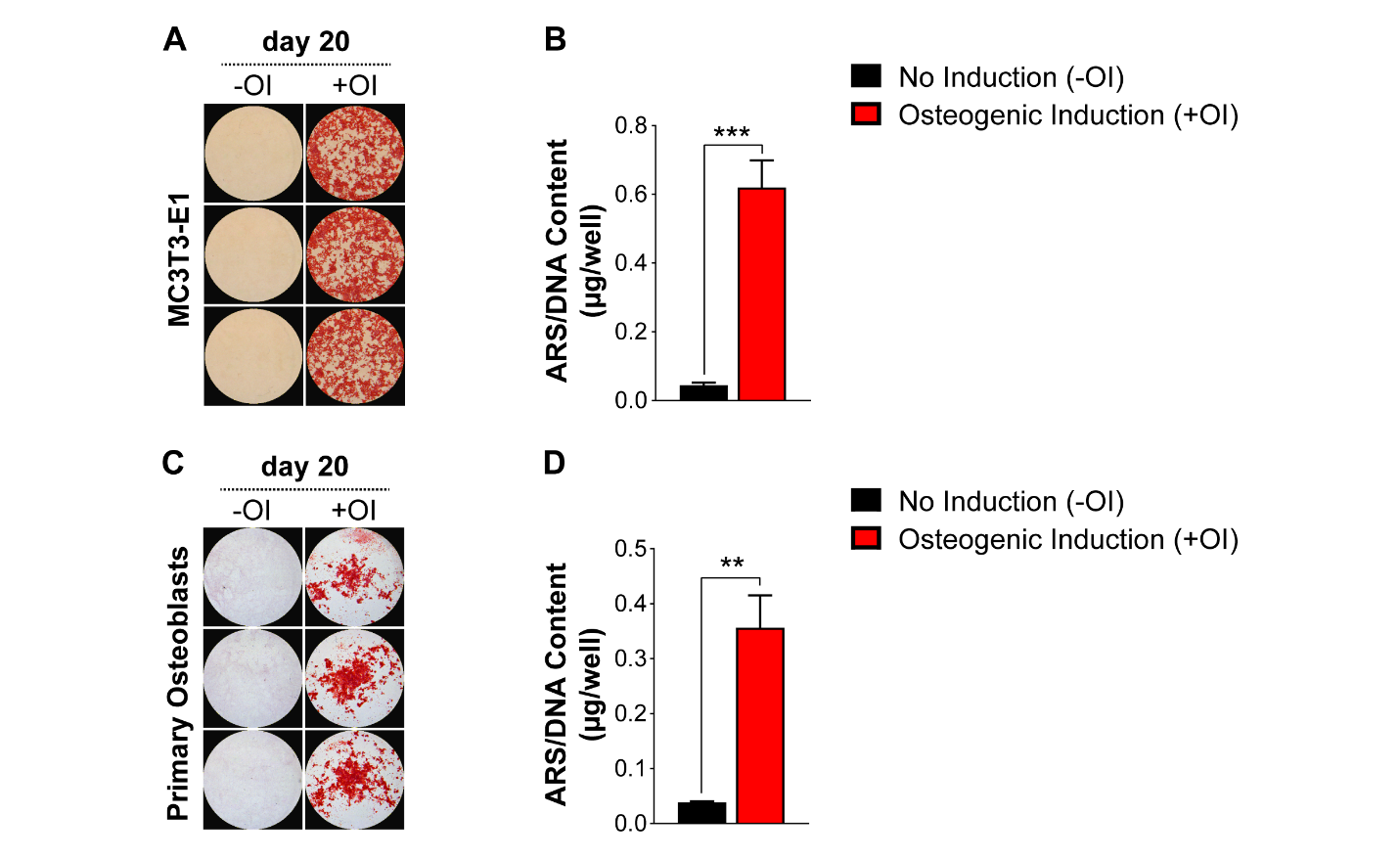


Supplementary Figure S2. Alizarin Red S (ARS) staining of MC3T3-E1 osteoblastic cell line and primary calvarial osteoblasts.

(A, C) ARS staining of MC3T3-E1 and primary osteoblasts in absence (-OI) or presence (+OI) of osteogenic induction medium at day 20. Both cell types were seeded in 24-well plate. (B, D) ARS quantification normalized to DNA content measured by Hoechst 33258 assay, in MC3T3-E1 cells (n=8) and primary osteoblasts (n=4). Data are expressed as mean ±SEM. **p* < 0.05, ***p* < 0.01, ****p* < 0.001.

**Supplementary Figure S3**


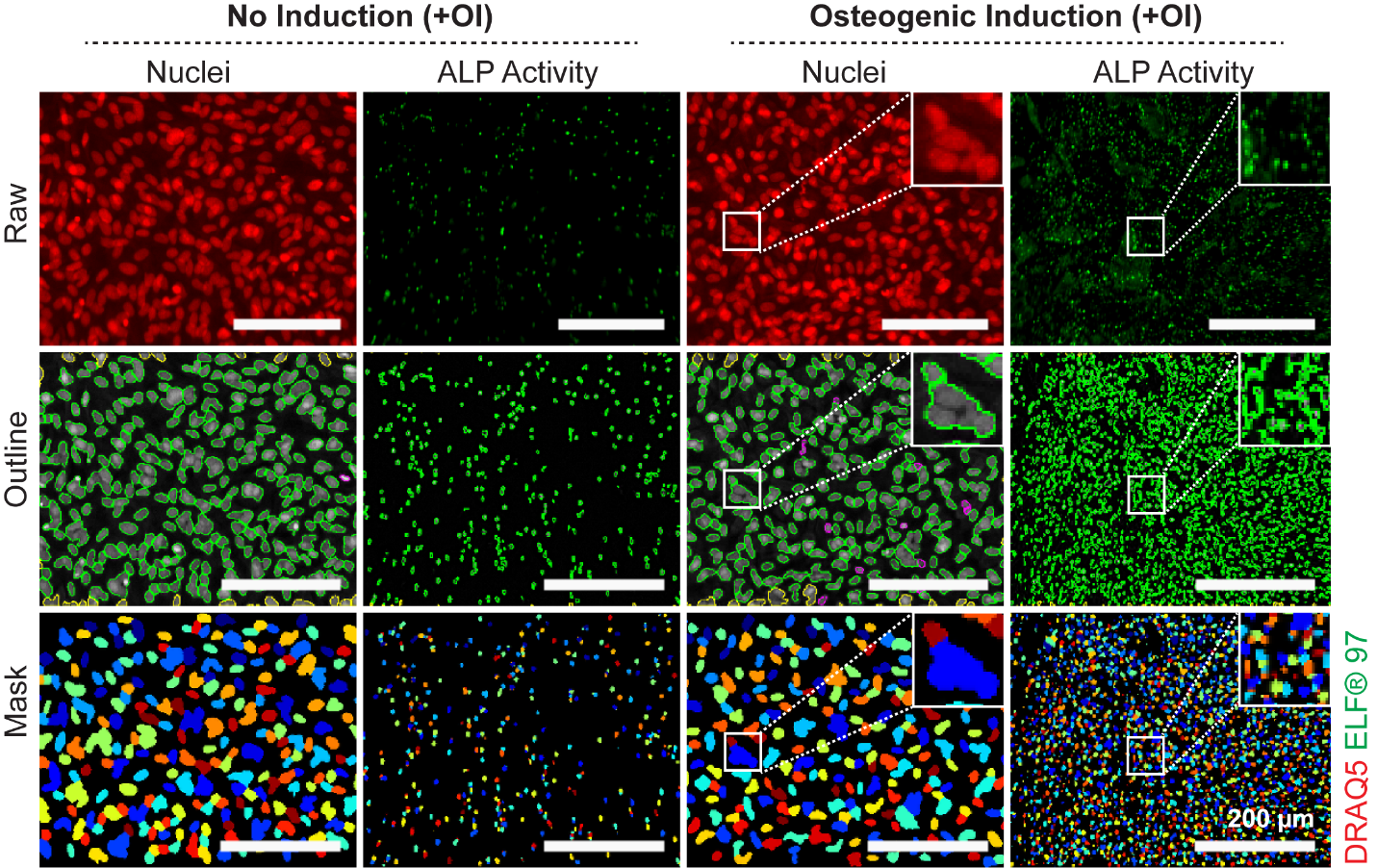


Supplementary Figure S3. Representative images of undifferentiated (-OI) and differentiated (+OI) MC3T3-E1 osteoblastic cell line and their cell identification and segmentation using CellProfiler.

(A) The upper panel show raw images of nuclei and alkaline phosphatase (ALP) staining of -OI and +OI condition in MC3T3-E1 cells, stained with DRAQ5 (red) and ELF® 97 (green) respectively. The middle panel depicts the outline of the nuclear and ALP staining of -OI and +OI MC3T3-E1 cells. The lower panel shows the segmentation of nuclei and ALP from -OI and +OI condition of MC3T3-E1 cells. The segmentation can be visualized by differences in colors between adjacent cells, in both nuclear and ALP channels (lower panel). Scale bar 200 μm.

**Supplementary Figure S4**


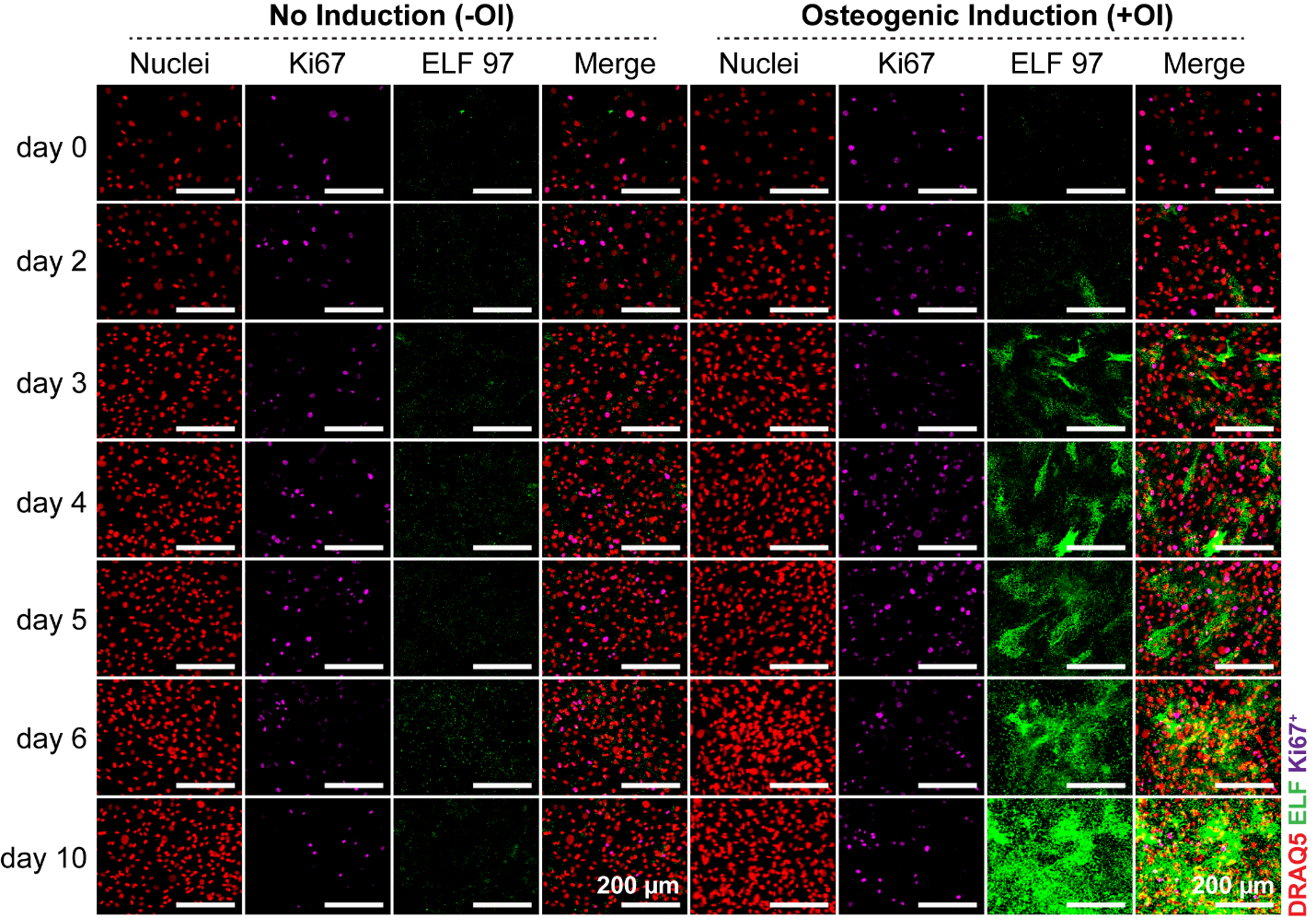


Supplementary Figure S4. DRAQ5, Ki67 and ELF® 97 staining at different stages of osteoblast differentiation in primary calvarial osteoblasts.

Representative images of DRAQ5, Ki67, and ELF® 97 staining from undifferentiated (-OI) and differentiated (+OI) cells at different time points. The cells were stained with DRAQ5 (red), Ki67 (purple), and ELF® 97 (green) for nuclear, proliferative and ALP staining respectively. Scale bar: 200 μm.

**Supplementary Figure S5**

**
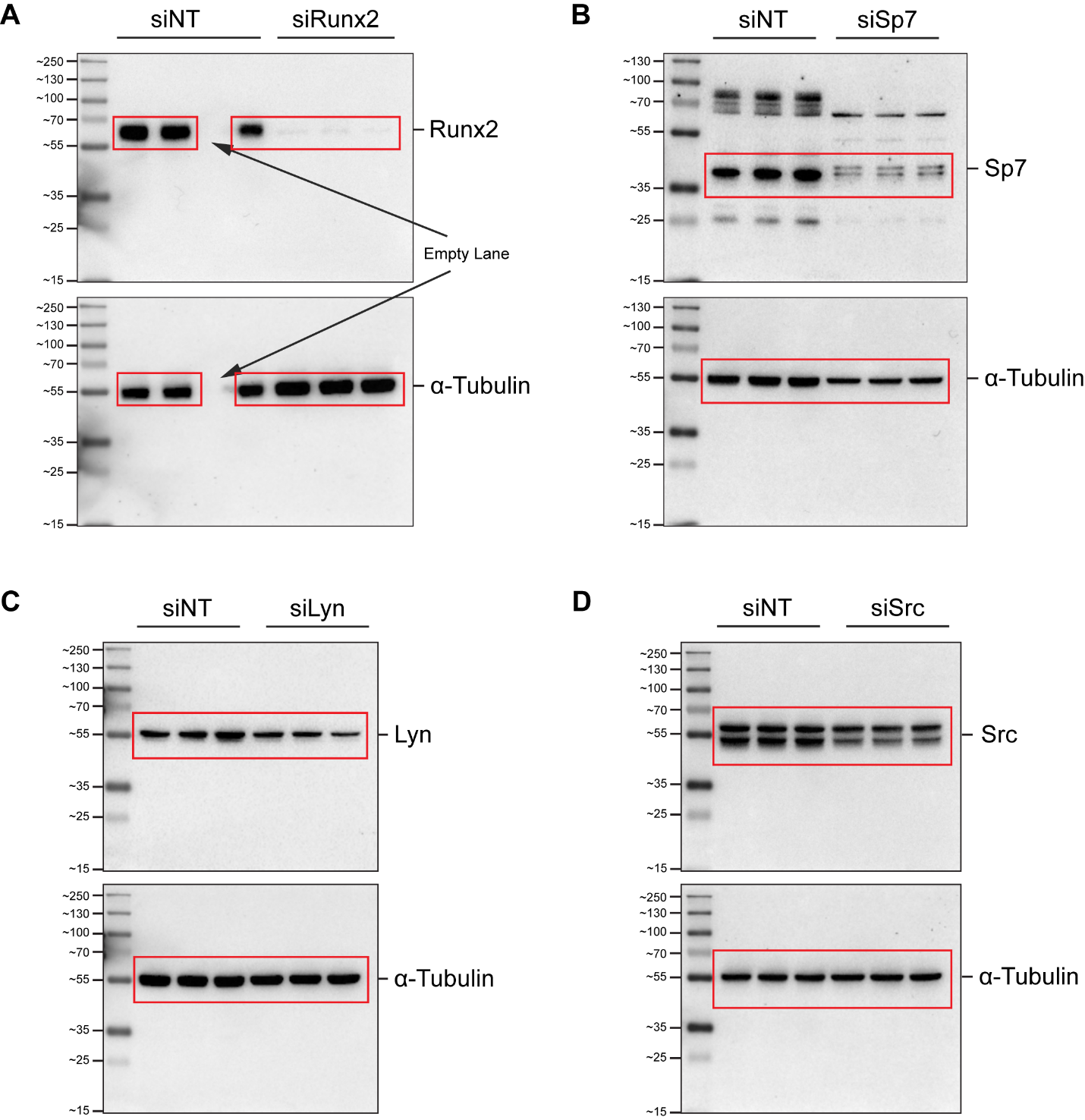
**

Supplementary Figure S5. Full blots related to figure 5.

Portions that have been chosen for presentation are marked in red (A) Full blots related to Figure 5E. Lane 3 is empty due to the damaged well (the empty lane has been cropped in the Figure 5E, shown with the dotted line). (B) Full blots related to Figure 5G. (C) Full blots related to Figure 5I. (D) Full blots related to Figure 5K.

Supplementary Table S1. siRNA transfection protocol for different plate formats.


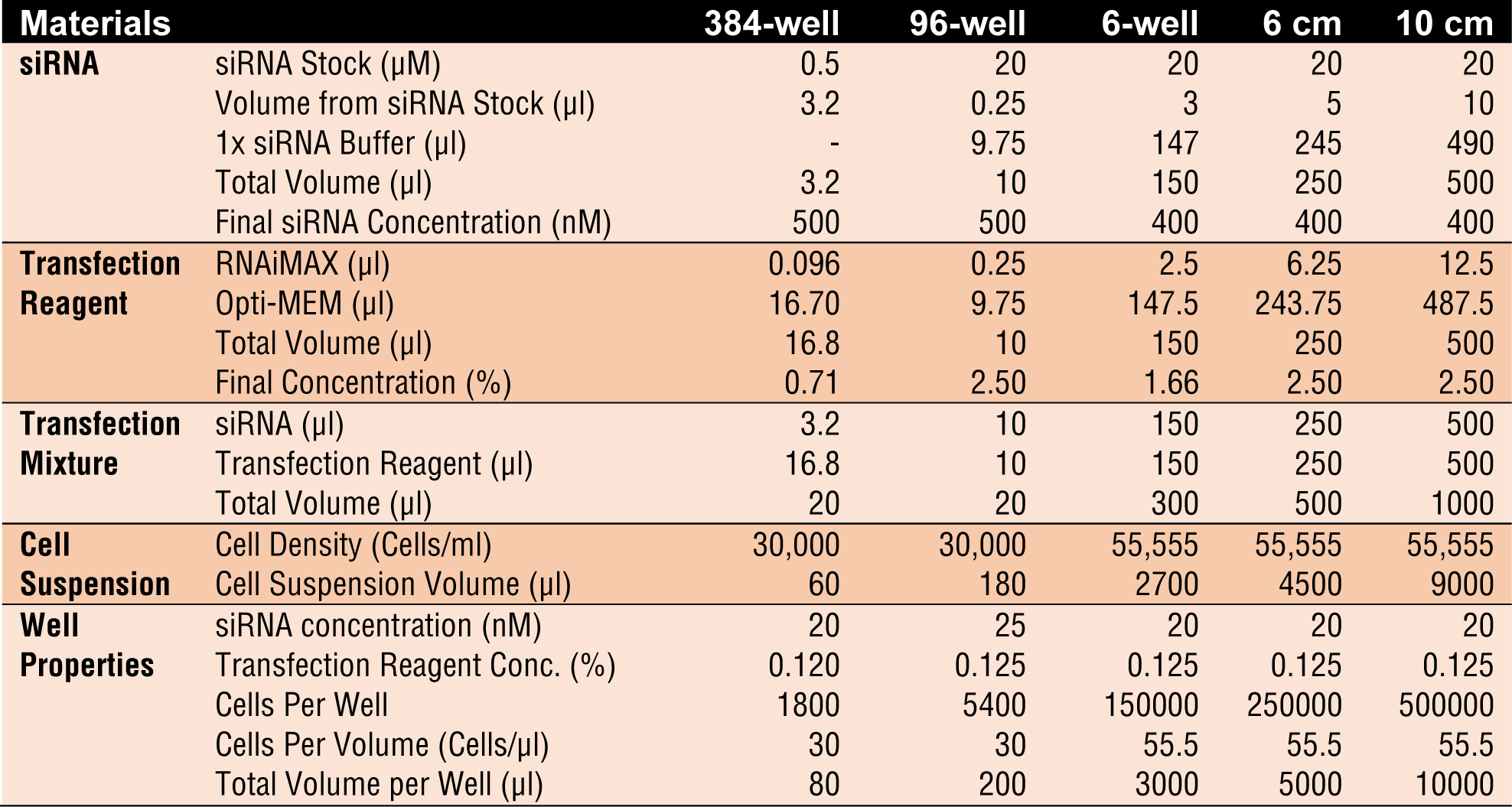


Supplementary Table S2. Oligonucleotide primer sequences used in real-time PCR.


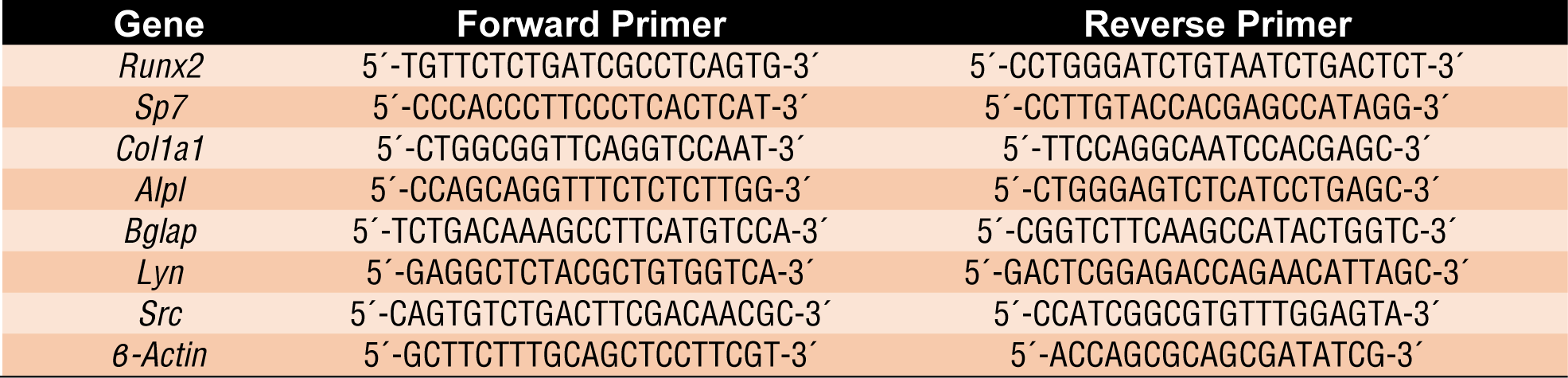

Supplement: Supplementary file 1 — Supplementary Information. [file 41598_2018_32364_MOESM1_ESM.docx]
